# Estimating the carbon budget and maximizing future carbon uptake for a temperate forest region in the U.S.

**DOI:** 10.1186/1750-0680-7-6

**Published:** 2012-06-19

**Authors:** Stith T Gower, Joseph Buongiorno

**Affiliations:** 1 Department of Forest and Wildlife Ecology and Management, University of Wisconsin, 1630 Linden Drive, Madison, WI, 53706, USA; 2 Present address: Department of Botany, University of Wyoming, 1000 E. University Ave, Laramie, WY, 82071, USA

**Keywords:** Carbon budget, Biome-BGC, Temperate forest, Forest harvest, Carbon sequestration, Life cycle inventory, Forest products

## Abstract

**Background:**

Forests of the Midwest U.S. provide numerous ecosystem services. Two of these, carbon sequestration and wood production, are often portrayed as conflicting. Currently, carbon management and biofuel policies are being developed to reduce atmospheric CO_2_ and national dependence on foreign oil, and increase carbon storage in ecosystems. However, the biological and industrial forest carbon cycles are rarely studied in a whole-system structure. The forest system carbon balance is the difference between the biological (net ecosystem production) and industrial (net emissions from forest industry) forest carbon cycles, but to date this critical whole system analysis is lacking. This study presents a model of the forest system, uses it to compute the carbon balance, and outlines a methodology to maximize future carbon uptake in a managed forest region.

**Results:**

We used a coupled forest ecosystem process and forest products life cycle inventory model for a regional temperate forest in the Midwestern U.S., and found the net system carbon balance for this 615,000 ha forest was positive (2.29 t C ha^-1^ yr^-1^). The industrial carbon budget was typically less than 10% of the biological system annually, and averaged averaged 0.082 t C ha^-1^ yr^-1^. Net C uptake over the next 100-years increased by 22% or 0.33 t C ha^-1^ yr^-1^ relative to the current harvest rate in the study region under the optized harvest regime.

**Conclusions:**

The forest’s biological ecosystem current and future carbon uptake capacity is largely determined by forest harvest practices that occurred over a century ago, but we show an optimized harvesting strategy would increase future carbon sequestration, or wood production, by 20-30%, reduce long transportation chain emissions, and maintain many desirable stand structural attributes that are correlated to biodiversity. Our results for this forest region suggest that increasing harvest over the next 100 years increases the strength of the carbon sink, and that carbon sequestration and wood production are not conflicting for this particular forest ecosystem. The optimal harvest strategy found here may not be the same for all forests, but the methodology is applicable anywhere sufficient forest inventory data exist.

## Background

Whole system (biological + industrial ecosystem) carbon budgets are required to identify opportunities to increase carbon sequestration, decrease carbon emissions, and answer the all important “systems sustainability” question: “How do we determine when we are getting too much of a good thing?” [1] Harvest is currently the primary disturbance agent for Upper Great Lakes forests, an important determinant of net ecosystem carbon (C) dynamics [2], and produces wood fiber needed by forest products and energy. The biological carbon cycle of the Upper Great Lakes forests is positive (i.e. they store carbon in the wood and/or soil) [3-5], while simultaneously producing >250 Mm^3^ of harvested wood, including >50% of the supply for composite wood products (i.e. oriented strand board (OSB) and plywood).

Most forest carbon cycle studies have focused on the biological system. Although recent studies have considered carbon stored in forest products [5], the biological and industrial forest carbon cycles are linked and the forest industrial ecosystem (harvest, transportation, processing, consumption, and disposal) is a significant user of fossil fuel [2]. For example, 94% of total emissions from a life cycle inventory for dimensional lumber was from the long transportation supply chain [2]. We conducted a whole-system (biological + industrial) carbon budget analysis of the Chequamegon-Nicolet National Forest (CNNF), a 615,000 hectare temperate forest region in Northern Wisconsin, U.S. The region is characterized by moderate tree species diversity, gently rolling terrain, short growing season, cold winters, and a well-developed forest products industry. Currently, approximately 1% of the land area in the study area is harvested annually, and it is managed for timber production and many other uses (i.e. recreation, wildlife habitat, etc.).

The forest biological ecosystem carbon cycle was simulated with Biome-BGC [6,7], an ecosystem process model with interacting carbon, nitrogen, and water cycles [6,8]. The model calculates net primary production (NPP), heterotrophic respiration (R_H_) and net ecosystem production (NEP = NPP – R_H_). The forest industrial ecosystem carbon cycle was simulated using a linear model built from life cycle inventories (LCIs) of forest and paper products developed for the Upper Great Lakes forests, including the CNNF [9-11], and regional forest product statistics [12]. The model tracks CO_2_-equivalent (CO_2_-eq) emissions associated with the harvest and use of wood C from the biological model. Annual whole-system carbon balance, or net system production (NSP), was computed as the difference between the net biome production (NBP, net ecosystem production for all forest stands in the CNNF) and total C from CO_2_ emissions from the forest industrial ecosystem. A quantitative approach to maximize the cumulative NBP (summed over time and space) was built with a dynamic model of managed forests [13], the computed NBP response functions of the CNNF hardwood and conifer forest ecosystems, and the current age-class distribution within the CNNF. This study had two main objectives: 1) To develop and apply an optimization methodology to compute the maximum C uptake by the CNNF for the next 100 years, and 2) Apply the whole-system modeling approach to answer the question: “Can forests sequester carbon and provide wood fiber for wood and paper products, and perhaps bioenergy feedstock?” and report the current biological, industrial, and forest system carbon balance.

## Results

**Figure 1:**
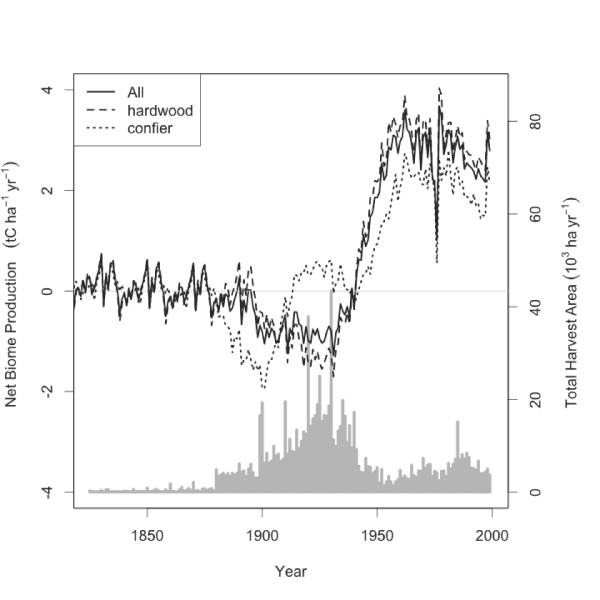
**Simulated carbon balance for the Chequamegon-Nicolet National Forest (CNNF), 1825–1999.** Spatial averages of net biome production, left-hand axis, are shown for the entire CNNF (All, solid line), hardwoods (large dash) and conifer (small dash). Positive values indicate a C sink relative to the atmosphere. Bar graph of simulated annual harvest area in CNNF, right-hand axis, is shown in light grey.

**Figure 2:**
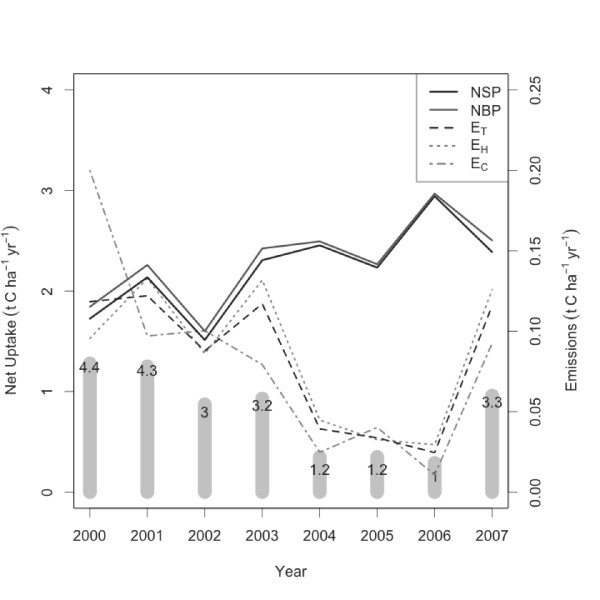
**Simulated carbon balance for the Chequamegon-Nicolet National Forest (CNNF), 2000–2008.** The left y-axis refers to the net system production (NSP) and forest net biome production (NBP) per unit of forest area. The right y-axis is scaled to the industrial forest C cycle emissions per unit of forest area for the entire CNNF (E_T_) and its hardwood (E_H_) and conifer (E_C_) components. Hardwood and conifer emissions are per unit area of each forest type. The bar plot (grey) shows harvest area (10^3^ ha) in each year.

Biome-BGC was run for years 1825–1999 (Figure 1) to simulate historical land use of the CNNF and past to near-present net biome production. The CNNF was a small C sink prior to the late 1800‘s when the majority of the forest was mature or old growth [14,15]. A well-documented, complete clear-cut harvest of the region’s forest occurred from 1880 to 1930 [14,15]. The CNNF transitioned from a C sink to a C source around 1900 and continued to be a C source until 1940 as a result of the region-wide harvest. Stand-killing disturbances, such as harvesting, cause negative net ecosystem production because heterotrophic respiration exceeds net primary production. Net biome production decreased as a greater fraction of the forest landscape consisted of recently disturbed forests. NBP recovered as the forest regenerated and harvest intensity decreased, and reached a maximum of 3.67 t C ha^-1^ (N = 6896 cells, excluding lakes, non-forested areas, etc.) in 1978 and averaged 2.72 t C ha^--1^ yr^-1^ from 1950 to 1999. Thereafter, NBP decreased (Figure 1) as expected by the stand age-related decrease in net primary production [16,17]. Mean simulated NBP for CNNF was 2.29 t C ha^-1^ yr^-1^ from 2000–2007, and ranged between 1.60 and 2.97 t C ha^-1^ yr^-1^ (Figure 2). NBP averaged 2.48 and 1.75 t C ha^-1^ yr^-1^ for hardwood (i.e. deciduous broadleaf) and conifer (i.e. evergreen needleleaf) forest types, respectively, but the CNNF average was closer to deciduous broadleaf forests because they comprised 75% of the CNNF.

Over the 8-year period (2000–2007, Figure 2), the industrial C budget for the CNNF averaged 0.082 t C ha^-1^ yr^-1^, and differed little between conifer and hardwood forests. The inter-annual variability in industrial ecosystem emissions was due to the inter-annual variability of harvested area, which ranged from 1,000 to 4,400 ha, and averaged 2,700 ha for the 8-year period (Figure 2). Between 2000 and 2007, industrial ecosystem emissions comprised from 1% to 7% of net system production.

Mean simulated net system production (NSP) was 2.21 t C ha^-1^ yr^-1^ (N = 6896 1 km^2^ cells, excluding lakes, urban areas, etc.), and ranged from 1.72 to 2.94 t C ha^-1^ yr^-1^ over the 8-year simulation period (Figure 2). This whole-system analysis illustrates that forests can store carbon — a valued ecosystem service — and still provide fiber for wood and paper products. This finding is important because these forests are “working forests”. The CNNF is likely representative for the Upper Great Lakes forests because they all share a similar past and current land use history and management practices and wood utilization are similar for private industry, non-industrial private landowners, and state forests [9,10].

**Figure 3:**
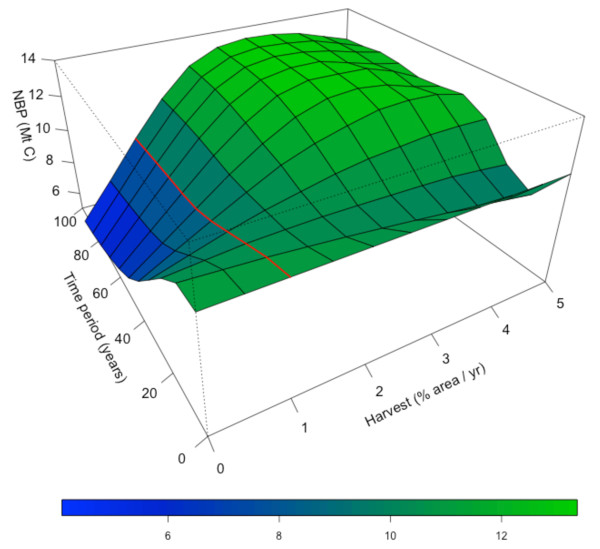
**Potential net C uptake for the CNNF in the next 100 years.** The x-axis is the percentage of the total area of the CNNF harvested yearly, held constant in every year. The y-axis is the time horizon, or number of 10-year increments. The red line shows NBP when annual harvest is held constant at 1% of the CNNF area.

**Figure 4:**
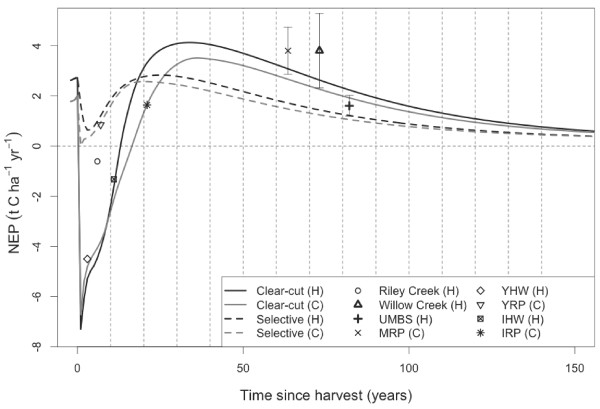
**Mean simulated response of net ecosystem production (NEP) to clear-cut (solid lines) and selective (dashed line) harvest over the entire simulation area, for both hardwood (black lines) and conifer (grey line) forest types.** Vertical dashed lines denote breakpoints for age-class calculations used in the biological C-budget optimisation. Points show annual estimates of NEP from eight eddy-covariance flux towers in Northern Wisconsin and Michigan in a harvest chronosequence [19], bold symbols denote site means values for sites with measurements in more than two years.

Over the next 100 years, simulated total NBP (i.e. cumulative C sequestered, excluding the industrial ecosystem) for the CNNF ranged from 78 to 136 Mt C (or 1.1 – 2.0 tC ha^-1^ yr^-1^) for constant annual harvest rates of 0- 5% of the total CNNF area (Figure 3). Current percentages of clearcut and selective harvests in CNNF (20 and 80%, respectively) were held constant throughout. The 1% harvest area scenario represents approximately 50% of the ‘Allowable Sale Quantity’ set by the CNNF forest plan [18]. Increasing harvest intensity temporarily decreased NBP, but increased NBP in the long term because a greater fraction of the forests were of optimal age for maximum NPP (Figure 4). We found that constant harvest or even a decrease in harvest does not maximize C uptake in the future (Figure 3). Net C uptake over the 100-year simulation period increased by as much as 22% or 0.33 t C ha^-1^ yr^-1^ relative to the 1% harvest area scenario when harvest intensity in each time period was allowed to vary between 0 and 2% (within the limits of the CNNF forest plan).

## Discussion

### Model evaluation and inference limitations

Comparison of our results to other studies is difficult, because to our knowledge no other whole system analysis of a forest landscape exists at this scale. However, simulated outputs of the biological C cycle agree with field measurements. Model outputs from Biome-BGC have been compared favorably to eddy-covariance and biometric data from sites within the CNNF region [20] (also see Figure 4). Biome-BGC captures the C dynamics associated with disturbances such as harvest [20,21] and fire [21,22] as evidenced by the good agreement between simulated and measured NEP for conifer and broadleaf deciduous forests of different ages [23-25] (Figure 4). In the historical reconstruction (1825–1999), simulated aboveground C recovered to roughly 60% of the pre-European settlement levels in 1999, in agreement with historical data from the region [26]. The average industrial C budget of 0.082 t C ha^-1^ yr^-1^ is consistent with the 0.06-0.12 tC ha^-1^ yr^-1^ reported by White *et al *[10]. Although we used some of the emission factors from that study in our industrial model, the model input (C harvested) was generated by Biome-BGC, whereas White *et al *[10] used reported harvest volumes.

Several sources of uncertainty are notable. We acknowledge the potential interaction between future climate, atmospheric CO_2_ concentration, and nitrogen deposition on both forest NBP and Biome-BGC model outputs in the next 100 years. Previous model simulations [22] and the results of the 1825–1999 simulation (Figure 1) both suggest that the effects of changes in climate and atmospheric chemistry are small compared to the “disturbance effect” in boreal and temperate regions in North America [22], further emphasizing the importance of this study. Stand-killing fire disturbance was the dominant driver of ecosystem C balance in the boreal zone of Central Canada [22] when compared to climate and atmospheric CO_2_. Although we did not test explicitly for past or future climate and CO_2_ effects, the extent of disturbance simulated in this study exceeded that in [22] (i.e. 1–2 stand-replacing disturbances at each model grid cell), thus it is likely that during the past 150 years the C balance in the CNNF has been dominated by human activity. In fact, had the large-scale harvesting not occurred, simulated NBP would be approximately zero (Figure 1, early years).

The study dealt only with the effects of forest harvest practice on the carbon balance for the CNNF, assuming that atmospheric CO_2_ and climate are stable over the time horizon considered. The effect of storms (i.e. tornados and wind) and insects on forest NBP were not considered here, however, large-scale stand-killing disturbances such as fire and wind are rare (> 400 year return interval) for Upper Great Lake forests [27]. Our estimate of NBP during and immediately following the cutover (1900–1940) may be slightly conservative (less of a C source), as it has been reported that logging residues were burned following the massive clear-cuts in the region [15], but there are insufficient data to simulate this over the entire CNNF. The harvest mass (C) output by Biome-BGC may not exactly match volumes extracted in harvest operations. The experiment was designed to match the spatial extent and harvest practices as described by CNNF foresters. Finally, in our calculation of NSP, we assume that the life cycle models for dimensional lumber, OSB, and magazine paper are similar on the CNNF as on non-federal forests [9,10]. These products do not represent the full spectrum of products manufactured from wood, but represent a large proportion of the total, averaging 89 and 99% for hardwood and conifer, respectively [28], and are the only data currently available at this scale. It is important to note that his analysis does not include C emission benefits associated with the substitution of forest products in energy and residential building systems. The benefits of forest management on net C balance may be enhanced when product substitution is included [29], especially as the time horizon considered increases.

### Sustainability and forest management policy implications

Although the specific results of this study may not be applicable to other forest regions with a different disturbance history and different response curves of NPP to stand-killing disturbance [16,17], the methodology for optimizing wood fiber production and carbon sequestration is widely applicable to sustainability science and carbon management. The current age structure, and hence, C sequestration and fiber production of the CNNF have been largely determined by human activities over the past century and a half [30-32]. Nevertheless, it is possible to maximize future C sequestration through strategic harvest. If forests are to continue to play a similar or greater role in supplying wood fiber for paper, wood, and biofuel feedstock, it is essential to educate forestland owners and managers, and policy makers about the trade-offs of managing forests for ecosystem goods and services. Our results suggest that working forests can sequester carbon and also provide wood fiber for wood and paper products. This finding has been corroborated in a similar analysis of a forest system in Europe [29].

Increased access to wood fiber from private and public forests will require educating the public that sustainable forestry is possible and can have environmental benefits. The US Forest Service’s management actions (e.g. harvesting) are greatly shaped by their perceived threat of litigation and defensibility in court, and the public and political groups’ understanding of the USFS proposed action [33]. Harvest actions were the primary source of the litigations by citizen groups concerned about the adverse effects of harvests on the environment, biodiversity, and other ecosystem services. The mangement objectives of non-industrial private landowners, the largest forest ownership group in the U.S. are influenced by more than net profit [34,35]. The increased demand for wood fiber, and perceived even greater demand for wood as a bioenergy feedstock [36] has increased harvesting in other regions, resulting in large transportation emissions and extreme greenhouse gas (GHG) emissions [2]. However, increased utilization of wood fiber from existing ecosystems to meet growing demand for food, fiber and bioenergy feedstock is not without possible environmental and socio-economic consequences. Notable concerns include degradation of long-term soil productivity due to increased nutrient removal, decreased biodiversity, and competition among wood, paper and bioenergy industries for a limited wood supply. The greater reliance on selective harvests, such as the scenario used in this study (Figure 3) greatly reduces the removal of nitrogen and carbon from soils [20]. Selectively harvested forests have many structural characteristics that resemble old-growth forests and therefore support higher biodiversity [37]. Stand structure affects the diversity of numerous fauna groups, including insects [38,39], birds [40,41], and small and large mammals [37]. This suggests that the dominant harvest type could have significant effects on biodiversity in the CNNF. In a meta-analysis study on the effects of forest thinning on biodiversity, Verschuyl *et al *[37] reported a positive or neutral response of mammal, breeding and wintering bird, amphibian, reptile, and insect species to thinning, but thinning intensity drove the magnitude of the response. Although forest thinning generally had neutral or positive effects on fauna groups, individual species of lepidoptra [39] and birds [41] were positively or negatively affected by changes in stand structure. Managing the CNNF for wood and paper products, carbon storage, and biodiversity are all critical components to sustainable forest management; optimizing one ecosystem good or service could have adverse effects on the others. Lastly, this study suggests the implementation of the optimized harvest scenario could increase wood fiber production, excluding any additional residue removal, and still maintaining current wood and paper product supply. A systems analysis of the biological and industrial ecosystems, such as the one outlined in this study,is a first step toward developing a sustainable natural resource and energy policy.

## Conclusions

Results of this modeling study suggest the CNNF forest system is currently a carbon sink, even when including carbon emissions from the industrial system. Ironically, the forest’s biological ecosystem current and future carbon uptake capacity is largely determined by forest harvest practices that occurred over a century ago, but we show an optimized harvesting strategy would increase future carbon sequestration, or wood production, by 20-30%, reduce long transportation chain emissions, and maintain many desirable stand structural attributes that are correlated to biodiversity. This study suggests the implementation of the optimized harvest scenario could increase both carbon sequestration and wood fiber production, excluding any additional residue removal, and still maintain current wood and paper product supply. A systems analysis of the biological and industrial ecosystems, such as the one outlined in this study, is a first step toward developing a sustainable natural resource and energy policy.

## Methods

### Biome-BGC process model

The biological forest C cycle was simulated using a spatially-explicit version of Biome-BGC that has been described previously [22,42]. Notable changes in this study were in the forest type determination and the location of harvested pixels. Instead of allowing dynamic competition between multiple vegetation types, we simulated only one cover type at each location, determined using data derived from the USFS forest inventory and analysis program (FIA) [43]. Biome-BGC was parameterized with average values [44] for deciduous broadleaf and evergreen needleleaf species for all parameters with the exception of specific leaf area (SLA) and the carbon to nitrogen ratio of leaves. As Biome-BGC is most sensitive to variation in SLA and C:N of leaves [44], we computed the mean value of all published values [44] for species within each FIA forest type rather than plant functional type averages. Whole-plant mortality fraction was set to 0.01, the average value for all tree species and age classes from a study conducted in the region [45]. Soil data were derived from the STATSGO2 database [46].

Model simulation started with the model self-initialization (or ‘spin-up’) routine. A spin-up simulation was run until the soil carbon pool reached equilibrium with climactic drivers. We assumed that the CNNF forests were in relative equilibrium with the atmosphere prior to 1800, and used the output from the spin-up simulation as initial conditions in 1800. We began our historical simulation in 1800, increasing atmospheric carbon dioxide and nitrogen deposition, and at the same time simulating the ‘cutover’ that occured in the CNNF region [14,15]. We simulated a clearcut harvest in every model cell at least once between the late 1800 s and early 1900s. Stands in the CNNF data that originated after 1950 were also harvested randomly between 1880 and 1950. Stands older than 1950 were only harvested once. We recognize that this methodology is not perfect, as a discrete land use history does not exist for every location in the study area. We have not included the effects of historic wildfire and the burning of harvest residues in this analysis that may have occurred in the early 1900s [15]. However, we aimed to make the best approximation the dominant disturbance and its timing over this region.

Our simulated harvest matched the area in the CNNF stand inventory data, using the year of stand origin as a surrogate for harvest year. Any pixels in the CNNF stand age data with unknown or missing age values (about 24% of the total) were estimated with data provided by the U.S. Forest Service Northern Research station (a change detection algorithm [47] applied to Landsat satellite data timeseries) for recent forest disturbance, or assigned an age by sampling randomly from the CNNF stand age distribution computed from the data. The area of conifer and hardwood forest type pixels summed to the area of harvest in the CNNF records, to the nearest square kilometer (for years 2000–2007). This was done for both clear-cut and selective harvest types, general descriptions of these harvests in the Biome-BGC context have been described previously [20].

The disturbance response functions (Figure 4) were computed using two additional 150-year simulations over the entire CNNF area, where either a clear-cut or a selective harvest was applied to every grid cell in the first year (2008) and a spatial average of NBP was computed annually. The effect of inter-annual variability in the 60-year climate data set on Biome-BGC outputs was removed using an ensembling method [21], CO_2_ and atmospheric nitrogen deposition were held constant at near-present day levels.

### Industrial ecosystem carbon cycle model

The industrial C cycle model was built using results from three published life-cycle assessment studies for forest products of the CNNF region: dimensional lumber [10,11], oriented strand board [10], and magazines [9]. Emission factors (t C emitted/t C processed) were 0.10 for harvesting operations, 0.11 for dimensional lumber (mean value of [10,11]), 0.11 for OSB, and 0.3 and for magazine paper production. Harvested C from the biological model entered one of three production chains and CO_2_-equivalent emissions associated with the production, transportation, use, and disposal of each product were computed. Carbon dioxide comprised > 99% of the total CO_2_-eq emissions [9]. The average proportion of the total harvest from the CNNF that entered each product chain was determined from the U.S. Forest Service’s timber products output database [12].

### Maximization of carbon uptake by the forest ecosystem

The potential C uptake for the forest ecosystem of the CNNF was computed by linear programming [13]. This methodology has been applied in forestry to maximize productivity, wood production, profit, etc. In this case, we extended it to net carbon uptake, utilizing spatial stand data for the CNNF and simulation outputs from Biome-BGC. The spatial data required were forest type and stand age, which is data commonly available for managed forests. For each forest type (or plant functional type, as we grouped the data in this analysis), data describing its NEP response as a function of time since harvest was also needed. For this analysis, we used Biome-BGC to compute the response function for each location in the CNNF, and computed an average for each forest type within 10-year age classes. Our decision to use 10-year age classes for the NEP response was based on prior experience with this methodology [13] and the need to create a practical tool which can compute the analytical solution rather quickly. The following model was applied to hardwood and conifer forests. The objective function was the total C uptake to be maximized over 100 years, from a forest described by 10 age-classes of 10 years each:

(1) $$\max z=\sum_{i=1}^{10}\sum_{j}^{10}c_{j}x_{\mathrm{ij}}$$

Where the decision variable, *x*_*ij*_, was the area harvested in decade *i* from age-class *j*, and *c*_*j*_ was the average NBP per unit area in age-class *j*, estimated with the response curves (Figure 4). The forest dynamics was described by:

(2) $$\begin{array}{l} a_{11}=a_{1}^{0},a_{21}=a_{21}^{0},\ldots ,a_{10,1}=a_{10,1}^{0}\\ a_{1j=}\sum_{i=1}^{10}x_{i}{}_{,j-1}\;j=2,\ldots ,11\\ a_{\mathrm{ij}}=a_{i}{}_{-1\text{,}\;j-1}-x_{i-1\text{,}\;j-1}\;i=2,\ldots ,9\text{;}\;j=2,\ldots 11\\ a_{10,11}=\left(a_{9,9}-x_{9,9}\right)+\left(a_{10,9}-x_{10,9}\right)\;j=2,\ldots ,11\\ x_{\mathrm{ij}}\geq 0,x_{\mathrm{ij}}\leq a_{\mathrm{ij}}\;i=1,\ldots ,10\text{,}\;j=1,\ldots 10 \end{array}$$

where *a*_*ij*_ was the area in age-class *i* at the beginning of decade *j*, and $a_{i1}^{0}$ was the initial area in age-class *i*. The following steady-state constraints

(3) $$a_{i,10}=a_{i,11}\;i=1,\ldots ,10$$

were used to ensure that the forest structure as the end of the 10th decade was the same as at the beginning, so that the harvest of the last decade could be continued in perpetuity. The total harvest area in any decade was constrained to not exceed a preset percentage of the total forest area:

(4) $$\sum_{i-1}^{10}x_{\mathrm{ij}}\leq \alpha \sum_{i-1}^{10}a_{i}^{0}$$

where α varied from 0 to 50% (0-5% area harvested annually per decade).

## Competing interests

The authors declare no competing interests.

## Authors’ contributions

S.T.G. and S.D.P. designed the experiment. S.D.P. assembled input data, wrote code, ran the model, and analyzed output data. J.B. and S.T.G. designed the optimization tool. S.T.G. administered the experiment and S.D.P., S.T.G, and J.B. wrote the manuscript. All authors read and approved the final manuscript.
